# Simulation of Exercise-Induced Syncope in a Heart Model with Severe Aortic Valve Stenosis

**DOI:** 10.1155/2012/138401

**Published:** 2012-12-05

**Authors:** Matjaž Sever, Samo Ribarič, Marjan Kordaš

**Affiliations:** ^1^Department of Hematology, Faculty of Medicine, University of Ljubljana, 1104 Ljubljana, Slovenia; ^2^Institute of Pathophysiology, Faculty of Medicine, University of Ljubljana, 1104 Ljubljana, Slovenia

## Abstract

Severe aortic valve stenosis (AVS) can cause an exercise-induced reflex syncope (RS). The precise mechanism of this syncope is not known. The changes in hemodynamics are variable, including arrhythmias and myocardial ischemia, and one of the few consistent changes is a sudden fall in systemic and pulmonary arterial pressures (suggesting a reduced vascular resistance) followed by a decline in heart rate. The contribution of the cardioinhibitory and vasodepressor components of the RS to hemodynamics was evaluated by a computer model. This lumped-parameter computer simulation was based on equivalent electronic circuits (EECs) that reflect the hemodynamic conditions of a heart with severe AVS and a concomitantly decreased contractility as a long-term detrimental consequence of compensatory left ventricular hypertrophy. In addition, the EECs model simulated the resetting of the sympathetic nervous tone in the heart and systemic circuit during exercise and exercise-induced syncope, the fluctuating intra-thoracic pressure during respiration, and the passive relaxation of ventricle during diastole. The results of this simulation were consistent with the published case reports of exertional syncope in patients with AVS. The value of the EEC model is its ability to quantify the effect of a selective and gradable change in heart rate, ventricular contractility, or systemic vascular resistance on the hemodynamics during an exertional syncope in patients with severe AVS.

## 1. Introduction

Exertional syncope, a syncope present during increased heart workload due to increased muscle work, is an important complication of advanced aortic valve stenosis (AVS) [1]. The mechanism of syncope is complex, and several factors contribute to its development [2]. What is common to all forms is an acute and brief reduction of cerebral blood flow leading to a transient loss of consciousness and followed by a spontaneous and complete recovery. The standard classification of the etiology of syncope recognizes three broad categories of pathophysiological mechanisms: the reflex (neurally mediated) syncopes, the syncopes due to orthostatic hypotension, and the cardiac (cardiovascular) syncopes [2].

A consistent change in hemodynamics during exertional syncope is systemic hypotension, a reduction in both systolic and diastolic blood pressure. Changes in heart rate are variable, ranging from insignificant to sinus bradycardia or even sinus node arrest [1–5]. Significantly, bradycardia does not occur simultaneously with a sudden fall of systemic arterial pressure but follows systemic and pulmonary hypotension [1, 3, 4]. Systemic hypotension during syncope can be the result of a reduced cardiac output, decreased peripheral resistance, or an inadequate venous return (e.g., due to venous pooling). Therefore, an exertional syncope in a patient with severe AVS can be either a reflex, a cardiac, or a combination of both. The reflex syncopes (RS) are a heterogeneous group of syncopes that attenuates the cardiovascular reflexes essential for the short-term control of adequate perfusion of vital organs. The effects of a RS are mediated by modulating the activity of the efferent sympathetic and parasympathetic pathways. When hypotension predominates, due to loss of vasoconstrictor tone, this syncope is subclassified as a vasodepressor reflex syncope (VRS). A cardioinhibitory variant (CIRS) is present when bradycardia or asystole are the predominating clinical signs. Therefore, the clinical signs can reflect a cardioinhibitory, a vasodepressor, or a mixed variant of the RS [2].

The triggers for the reflex syncope are not completely understood [6–8]. Most of the evidence does not support the suggestion that vasodilatation and bradycardia are triggered by a paradoxical stimulation of the cardiac ventricular receptors [7, 9–12]. More likely, the precise trigger for the vasovagal syncope varies from patient to patient and depends on a complex interaction of neuronal inputs, autonomic output, humoral effects, and ischaemia [7].

Cardiac syncopes can be due to arrhythmias (drug induced, idiopathic, or associated with myocardial ischemia/infarction) or due to structural diseases of the heart or major blood vessels. The most common cause of a cardiac (cardiovascular) syncope is an arrhythmia, for example, a bradycardia or a tachycardia [2].

Patients with severe AVS are more prone to exertional syncope than healthy individuals. Firstly, severe AVS limits the ability of the cardiovascular system to maintain an adequate perfusion of the brain during increased muscle work. Secondly, these patients may have developed compensatory changes (myocardial fibrosis and impaired coronary vasodilator reserve) that increase the chances of left ventricular dysfunction with or without myocardial ischemia or arrhythmias (bradycardias or tachycardias) during increased muscle work. To summarise, exertional syncope in a patient with severe AVS is caused by different mechanisms [7] and concomitant AVS-related structural changes of the cardiovascular system [13] that prevent an optimal adjustment of stroke volume, heart rate, or peripheral resistance to provide adequate perfusion of the brain.

We studied the differential effects of arrhythmia (e.g., bradycardia) or reduced stroke volume (e.g., decreased inotropy due to myocardial ischemia) in the presence of syncope-related homeostasis failure of the cardiovascular system with venous pooling of blood and decreased peripheral resistance. The lumped-parameter computer model of this cardiovascular system was based on equivalent electronic circuits (EECs). For the presented study we upgraded an existing EEC model of the cardiovascular system with AVS [14–16]. The upgraded model, for simulating exertional syncope in a patient with AVS, includes

1. an improved subcircuit controlling ventricular contractility that gives an ejection fraction value of 70% for a normal heart;
2. a heart model with severe AVS, moderately decreased left ventricular contractility, and systolic insufficiency (as a complication of compensatory ventricular hypertrophy). The simulated left ventricle still has a preserved but limited capacity to respond to inotropic mechanisms, thus more closely resembling the clinical conditions of a patient with AVS that develops exertional syncope;
3. the ability to simulate different patterns of the RS during exercise by selectively activating or inactivating the vasodepressor reflex (loss of venoconstriction) or the cardioinhibitory reflex (reduced left ventricular contractility or reduced heart rate).

In addition, the results of the simulation were analyzed with the pressure-volume (P-V) loop diagrams of left ventricular work.

## 2. Methods

Analyses are performed by developing an equivalent electronic circuit (EEC) by using Electronics Workbench (EWB) Personal version 5.12 [14–16]. In this model of the cardiovascular system, venous tone, heart rate, and contractility of the right and left ventricle are modulated by a negative feedback mechanism.

**Figure 1:**
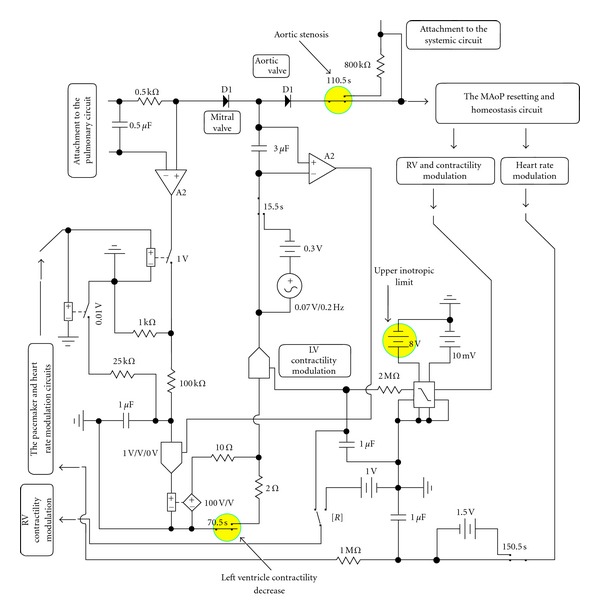
Equivalent circuit of the left ventricle. If left ventricular contractility is decreased (note the switch activated at 70.5 s), it is still acted upon by some residual inotropic influence. Note that its upper limit is determined by the battery in the voltage limiter. Aortic stenosis is simulated by introducing a series resistance 0.8 U (800 kΩ) at the output of the aortic valve.

Compared to the earlier EECs [14–16], the left ventricular circuit is slightly modified. The mitral and the aortic valves are simulated by diodes D1; therefore, in the left ventricle, there is no “reverse flow” (neither from aorta into the ventricle during diastole nor from the ventricle into the atrium during systole). The nominal gain is increased from 50 to 100, and in the left ventricle contractility modulation, the upper inotropic limit is increased up to 8 FGU (factor of gain units; 8 V; Figure 1).

Decreased left ventricle contractility is achieved by decreasing the nominal gain from 100 to about 20 (Figure 1). AVS is simulated by increasing output resistance of the ventricle (0.8 U) by inserting a 800 kΩ resistor in series to the output diode. Please note that the unit to measure the resistance to flow (U) is defined as 100 mm Hg/100 mL/s (For details please refer to [14–16]).

Results are shown graphically, as the time course of equivalent variables. Thus, electrical variables: voltage, current, resistance, capacitance, and charge, correspond to physiological variables: pressure, blood flow, resistance, capacitance, and volume (for details refer to [14–16]). The interdependence of pressure and volume of the left ventricle is shown by pressure-volume analysis (P-V loop diagrams) describing the left ventricle work load during one cardiac cycle. Acronyms of variables studied are listed in Table 1.

## 3. Results

**Figure 2:**
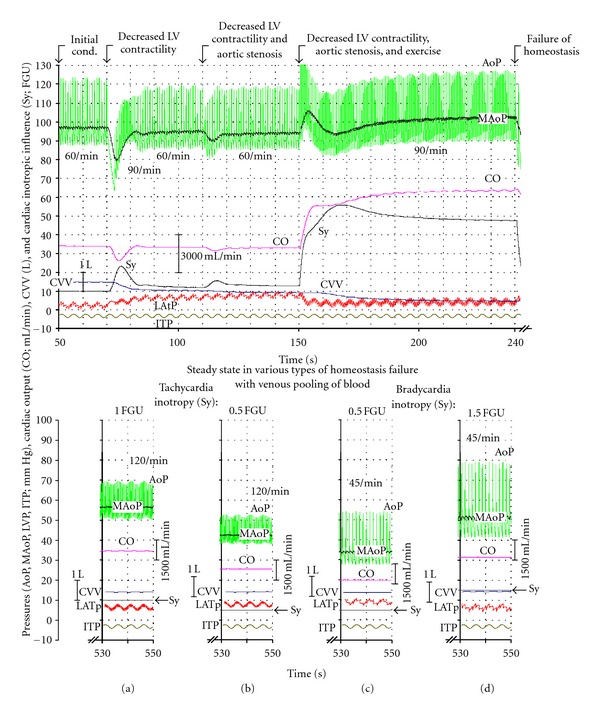
Top records. The time course of AoP, MAoP, CO, CVV, Sy, and LAtP in initial conditions (50 s–70.5 s), after LV contractility is decreased (70.5 s–110.5 s), after aortic stenosis (110.5 s–150.5 s), after exercise (150.5 s–240.5 s), and after failure of homeostasis (240.5 s onwards). If a parameter is changed, due to the homeostatic negative feedback, a transient phenomenon shows up, but very soon a new steady state is established. As expected, exercise (decreased peripheral resistance and MAoP reset) results in an increased Sy, and consequently, increased heart rate, CO, MAoP, and systolic AoP. Steady state levels of variables, depending on the type of homeostasis failure, are shown in the bottom records in columns (a–d) (530 s–550 s of simulation time). Failure of homeostasis results in a dramatic change of almost all variables. Steady state levels of variables (interval 530 s–550 s of simulation time) depend on the type of homeostasis failure. At heart rate 120/min and Sy equal to 1 FGU, AoP is decreased and pulse pressure lowered (a). A decrease in Sy results in a further decrease of AoP and MAoP (b). At a heart rate of 45/min and a Sy equal to 0.5 FGU, the AoP and MAoP are extremely low (c). If the Sy is increased to 1.5 FGU, the MAoP is low and the pulse pressure very large (d).

**Figure 3:**
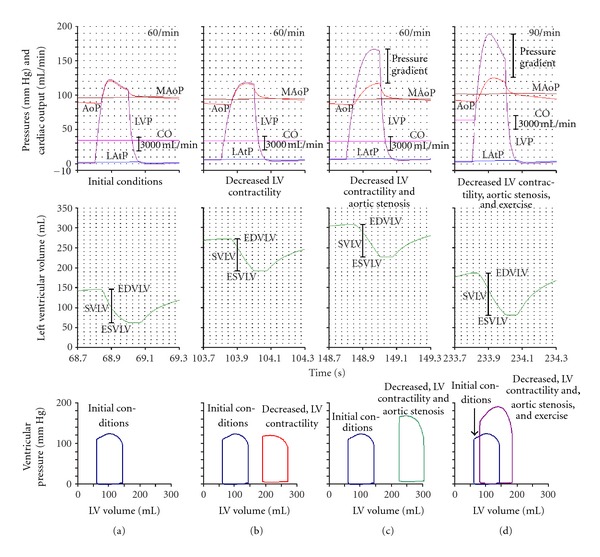
Data are arranged in four columns according to the simulation sequence described in Figure 2. In the upper parts of each column, there are time courses of various variables during systole and part of diastole (AoP, MAoP, LVP, LAtP, CO, ventricular volume). Note that a decreased LV contractility results in a decreased rate of contraction of LV (b). After aortic stenosis, a ventriculoaortic pressure gradient appears (c). EDVLV is increased, but due to a negative feedback SVLV is almost normal. In exercise (d), the pressure gradient is further increased, but due to the negative feedback (Sy increased) the heart rate and CO are increased and the EDVLV decreased. These changes are in excellent agreement with corresponding P-V loop diagrams. Note that in decreased LV contractility, the P-V loop diagram is shifted to the right. In aortic stenosis (note the pressure gradient), due to the inotropic effect (increased Sy), the P-V loop diagram shows a large ventricular pressure. The full inotropic effect is shown in exercise, where the P-V loop diagram is shifted to the left.

In all simulations, the sequence of parameter change (in the five time sequence sections) is as follows:

1. 50 s–70.5 s: sedentary, normal (initial) conditions;
2. 70.5 s–110.5 s: development of decreased LV contractility in sedentary conditions;
3. 110.5 s–150.5 s: development of decreased LV contractility and severe AVS in sedentary conditions;
4. 150.5 s–240.5 s: decreased-LV contractility and AVS, and MAoP reset and peripheral resistance decreased by 50% during physical exercise;
5. 240.5 s–550 s: failure of homeostasis due to activation of the reflex syncope response during physical exercise (during exertional syncope). This is achieved by differentially switching off the negative feedback circuits controlling heart frequency, left ventricular contractility, and venous tone. Figures 2 and 3 show hemodynamic changes after activation of a RS response with inhibition of left ventricular contractility and vasodilatation but no significant effect on heart frequency.

The time course of AoP, MAoP, CO, CVV, Sy, and LAtP during simulation as described above is shown in Figure 2, top records. Note that, if a parameter is changed, a transient phenomenon shows up, but very soon a new steady state is established. Thus, after LV contractility is decreased, AoP, MAoP, and CO are transiently decreased and heart rate increased. However, due to the homeostatic negative feedback, Sy is increased, resulting also in venoconstriction and decreased CVV. Therefore, LAtP is increased. Similar changes, also compensated by Sy, can be seen after aortic stenosis is induced. As expected, exercise (decreased peripheral resistance and MAoP reset) results in an increased Sy, and consequently, increased heart rate, CO, MAoP and systolic AoP. 

Failure of homeostasis results in a dramatic change of almost all variables; their steady state is established at about 500 s of simulation time (Figure 2, bottom records). However, steady state levels of variables depend on the type of homeostasis failure. If heart rate is 120/min and Sy is 1 FGU, CO is almost equal to initial conditions despite hypotension and small pulse pressure (Figure 2(a)). A decrease in Sy results in a decreased CO as well as AoP and MAoP (Figure 2(b)). If heart rate is 45/min and Sy is 0.5 FGU, CO, AoP, and MAoP are extremely low (Figure 2(c)). However, if Sy is increased to 1.5 FGU, CO is almost equal to initial conditions, but MAoP is low and pulse pressure very large (Figure 2(d)).

The time course of AoP, MAoP, LVP, LAtP, and CO during one cardiac cycle and the corresponding P-V loop diagram, are shown in Figure 3, in four sections (columns; cf. Figure 3).

1. Normal, sedentary (initial) conditions (first column; time sequence 68.7 s–69.3 s): note that peak LVP is reached in less than 0.1 s, indicating a relatively high rate of LV contraction. SVLV is 87 mL, EF is 63%, and diastolic LVP is slightly negative. Accordingly, the P-V loop diagram shows relatively low ventricle volume and low ventricular diastolic pressure.
2. Decreased LV contractility in sedentary conditions (second column; time sequence 103.7 s–104.3 s): maximum LVP is almost normal, but peak LVP is reached in more than 0.1 s, indicating a relatively low rate of LV contraction. EDVLV and ESVLV are strongly increased, SVLV only slightly decreased, but EF strongly decreased to about 30%. Diastolic LVP is positive. Accordingly, the P-V loop diagram is shifted to the right, to an increased ventricular diastolic pressure and a relatively high ventricle volume.
3. Decreased LV contractility and AVS in sedentary conditions simulate the conditions of an AVS related systolic ventricular dysfunction (third column; time sequence 148.7 s–149.3 s): an aortic-ventricular pressure gradient (about 50 mm Hg) shows up. Maximum LVP is almost 170 mm Hg, but peak LVP is reached in more than 0.1 s, indicating a relatively low rate of LV contraction. EDVLV and ESVLV further increased, SVLV maintained, and EF strongly decreased to about 25%. The P-V loop diagram is shifted strongly to the right and upwards, to a relatively very high ventricle volume and systolic pressure.
4. Decreased LV contractility AVS and MAoP reset, and peripheral resistance decreased by 50% during physical exercise (fourth column; 233.7 s–234.3 s): the aortic-ventricular pressure gradient is increased to about 70 mm Hg. Maximum LVP is almost 190 mm Hg and peak LVP is reached in about 0.1 s, indicating an improved rate of LV contraction. EDVLV and ESVLV decreased, SVLV is about 107 mL, and EF 56%. Accordingly, the P-V loop diagram is shifted to the left and upwards, to a relatively low ventricle volume and high ventricle systolic pressure.

**Figure 4:**
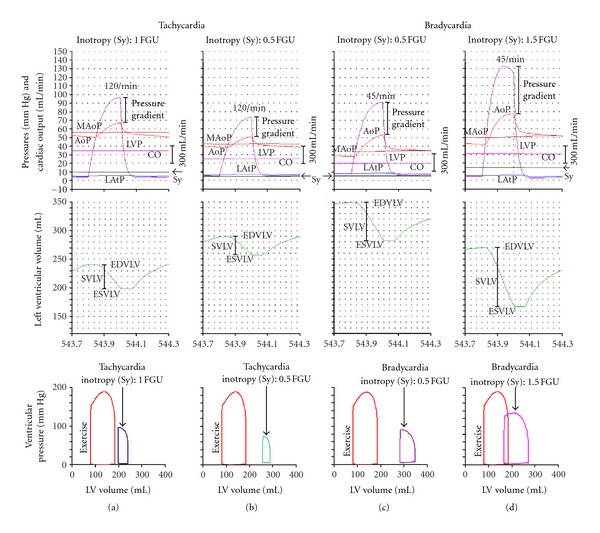
Data are arranged in four columns according to the type of homeostasis failure, after the steady state is established. The upper two sections of columns (a–d) present the time course of AoP, MAoP, LVP, LAtP, ventricular volume, and CO during systole and part of diastole (543.7 s–544.3 s). Note that the pressure gradient and its magnitude are dependent on the Sy and on the heart rate. In the bottom of each column there are the corresponding P-V loop diagrams. In these, for the sake of comparison, also the P-V loop diagram in exercise is shown (cf. Figure 3(d)). If the heart rate is 120/min and the Sy is 1 FGU 4(a), the P-V loop diagram is depressed, shifted to the right, and narrowed. If in this condition Sy is decreased (0.5 FGU; 4(b)), these changes are even more pronounced. They agree excellently with the corresponding time course of various variables, showing the decreased LVP and SVLV and the increased EDVLV. If the heart rate is 45/min and the Sy is 0.5 FGU, the P-V loop diagram is shifted to extreme right, but maintains its width. EDVLV is strongly increased, increasing also SVLV. If in this condition the Sy is increased to 1.5 FGU, the P-V loop diagram is shifted to the left, increasing its width: the EDVLV is decreased, but the SVLV is increased, as is also shown in the time course of ventricular volume and pressure.

Various types of homeostasis failure are also shown in steady state (after about 540 s of simulation time) as the time course of AoP, MAoP, LVP, LAtP, and CO during systole and part of diastole (543.7 s–544.3 s). All data are shown in columns (a)–(d) (Figure 4, cf. also Figure 2), including the corresponding P-V loop diagrams. In these, for the sake of comparison, also the P-V loop diagram in exercise is shown (cf. Figure 3(d)). Note that the pressure gradient and its magnitude are dependent on Sy and on heart rate.

If heart rate is 120/min and Sy is 1 FGU (Figure 4(a)), the P-V loop diagram is depressed, shifted to the right, and becomes narrow. If in this condition Sy is decreased (0.5 FGU; Figure 4(b)), these changes are even more pronounced. They agree excellently with corresponding time course of various variables, showing decreased LVP, SVLV, and increased EDVLV. 

If the heart rate is 45/min and the Sy is 0.5 FGU, the P-V loop diagram is shifted to extreme right, but maintains its width. EDVLV is strongly increased, increasing also SVLV. If in this condition the Sy is increased to 1.5 FGU, the P-V loop diagram is shifted to the left, increasing its width: EDVLV is decreased, but SVLV increased, as is also shown in the time course of ventricular volume and pressure.

## 4. Discussion

### 4.1. General and Technical Comments

Technical details important to be considered in simulations have already been discussed [14]. Note that gain in left ventricle circuit is increased from 50 to 100. Therefore, the left ventricle contractility (and its ejection fraction) is improved. Therefore, blood volume is slightly smaller.

Compared to earlier AVS simulations [14], in the present EEC, the left ventricle contractility simulation is upgraded similarly as in [16]. Therefore, if LV basic contractility is decreased (Figure 1; nominally by about 80%), it can be, to a limited degree, still acted upon through the inotropic mechanism of the negative (homeostatic) feedback.

It should be noted that at any heart rate, the duration of systole is constant, 0.2 s. Therefore, if heart rate is increased, only the duration of systole is decreased. Atria do not contract. It should also be noted that in the present circuit:

1. a flow-dependent decrease in pulmonary vascular resistance is not simulated;
2. the control of peripheral (arteriolar) resistance is not included into the negative feedback.

In principle, it would be possible to include both features. However, this would contribute considerably to the complexity of the circuitry, without contributing very much to the understanding of underlying physiological mechanisms.

Despite the model's deficiencies described above, the negative feedback (incorporating the control of venous volume, of contractility of RV and LV, and of heart rate) seems to be quite similar to that controlling the human cardiovascular system [17–19]. After a parameter in the circuit is changed (i.e., a disturbance introduced), the time course of many variables can be studied to demonstrate the relation—not only on a magnitude scale, but also on a time scale—between the disturbance and homeostatic response. The changing time course of left ventricle pressure also shows how total vascular impedance—affected by AVS and by peripheral vasodilatation—affects the process of left ventricle contraction.

By using the present model various physiological and clinical conditions have been simulated (effects of heart rate changes, volume loading, exercise, heart failure, AVS, mitral and aortic regurgitation). Our unpuplished data also show that, by a suitable modification of the ventricle circuit, even the cardiac aneurysm can be simulated quite satisfactorily. 

### 4.2. Comparison with Other Simulations

In the present EEC, blood inertia is not simulated; therefore, the aortic dicrotic notch—as simulated in [20]—is absent. Changes effected by left ventricular failure were simulated by Kim et al. [20]. In their work, results were shown as a time course of left ventricular pressure and of left ventricular volume and also as P-V loop diagrams. All data are, qualitatively, readily comparable to data obtained by the presented EEC of exertional failure with AVS. However, some minor quantitative differences can be observed, probably due to the fact that in the presented EEC, homeostatic (feedback) mechanism(s) is (are) operative until their failure is simulated due to the activation of the RS response during exercise.

Hemodynamic changes effected by AVS were simulated by Korürek et al. [21]. This model also simulated atrial contraction. Results were shown as the time course of left atrial and ventricular pressure and of left ventricular volume and as ventricular P-V loop diagrams. All data are, qualitatively, readily comparable to data obtained by the presented EEC. However, some quantitative differences can be observed, probably due to the fact that in our EEC, the simulation protocol is different. Our model simulates the physiological negative pressure in the thoracic cavity. Therefore, after vigorous left ventricular contraction, diastolic ventricular pressure may become negative. Furthermore, prior to AVS, left ventricular contractility is decreased, and the homeostatic (feedback) mechanism(s) is (are) operative until the onset of simulated exertional syncope when they are differentially inhibited by the vasodepressor or cardioinhibitory component of the RS response.

Compared to the simulations described above [20, 21], the present EEC allows simulations exactly as in animal experiment. Not only transient phenomena and steady state of various variables can be recorded, but also the rate and magnitude of the homeostatic response can be studied.

### 4.3. Comparison of the EEC Exertional Syncope Model with Patient Data

There are only a few published reports of exercise-induced syncope in patients with aortic stenosis [1, 3–5]. Consistently, the reports show a reduction in systemic arterial pressure, due to a reduction in both systolic and diastolic blood pressure. Changes in heart rate are variable, ranging from insignificant to tachycardias, bradycardias, or even to a cessation of ventricular contractions. What is important is that, during a RS, hypotension precedes bradycardia [8], thus ruling out the explanation that RS is essentially due to activation of the baroreceptor reflex. Patient with AVS can develop arrhythmias before, during, or after an exercise-induced syncope. However, arrhythmias are not essential for the development of exercise-induced syncope [3]. The exertional syncope of patients with AVS was reported to be associated with ECG signs of myocardial ischemia [1, 4, 5] but also occurred in the absence of left ventricular failure [3].

Our model demonstrates the changes in hemodynamics (a reduction in both systolic and diastolic blood pressure) when the capacity of the heart to maintain a sufficient cardiac output is reduced either due to arrhythmia or to reduced contractility. Therefore, the results of the model are qualitatively in agreement with the published data [1, 3–5]. Since clinical data are available at the level of case series, any statistical comparison with the model is not possible and the results should be evaluated in a descriptive way. The value of the EEC model is its ability to quantify the effect of a selective and gradable change in heart rate, ventricular contractility, or systemic vascular resistance on the hemodynamics during an exertional syncope in patients with severe AVS.

## 5. Conclusions

The presented lumped-parameter EEC model can differentially simulate the effect of cardioinhibitory and vasodepressor component of the RS response, and the simulated hemodynamic data are in agreement with patient data recorded during exercise-induced syncope. To our knowledge, this is the first reported computer simulation of an exercise-induced RS in patients with severe AVS.

## Figures and Tables

**Table 1:** Recorded variables (with corresponding units) and acronyms used in text and illustrations.

Variable	Acronym
Aortic pressure (mm Hg)	AoP
Cardiac output (mL/min)	CO
Contractible volume of veins (mL)	CVV
Ejection fraction of left ventricle	EF
End-diastolic pressure in left ventricle (mm Hg)	EDPLV
End-diastolic volume of left ventricle (mL)	EDVLV
End-systolic volume of left ventricle (mL)	ESVLV
Factor of gain (FGU)	FG
Intrathoracic pressure (mm Hg)	ITP
Left atrial pressure (mm Hg)	LAtP
Left ventricle	LV
Left ventricular pressure (mm Hg)	LVP
Left ventricular volume (mL)	LVV
Mean arterial pressure (mm Hg)	MAoP
Stroke volume of the left ventricle (mL)	SVLV
Sympathetic (inotropic) homeostatic contractility modulation	Sy
